# Cardiovascular benefits of *Eruca sativa* mill. Defatted seed meal extract: Potential role of hydrogen sulfide

**DOI:** 10.1002/ptr.7479

**Published:** 2022-04-27

**Authors:** Lara Testai, Eleonora Pagnotta, Eugenia Piragine, Lorenzo Flori, Valentina Citi, Alma Martelli, Lorenzo Di Cesare Mannelli, Carla Ghelardini, Roberto Matteo, Serafino Suriano, Antonio Troccoli, Nicola Pecchioni, Vincenzo Calderone

**Affiliations:** ^1^ Department of Pharmacy University of Pisa Pisa Italy; ^2^ Interdepartmental Research Center Nutrafood “Nutraceuticals and Food for Health” University of Pisa Pisa Italy; ^3^ Interdepartmental Research Centre of Ageing Biology and Pathology University of Pisa Pisa Italy; ^4^ CREA‐Council for Agricultural Research and Economics, Research Centre for Cereal and Industrial Crops Bologna Italy; ^5^ Department of Neuroscience, Psychology, Drug Research and Child Health–Neurofarba– Pharmacology and Toxicology Section University of Florence Florence Italy; ^6^ CREA‐Council for Agricultural Research and Economics, Research Centre for Cereal and Industrial Crops Foggia Italy

**Keywords:** cardiovascular system, *Eruca sativa* mill., erucin, glucoerucin, hydrogen sulfide

## Abstract

*Eruca sativa* Mill. is an edible plant belonging to the *Brassicaceae* botanical family with a long story as a medicinal material, mainly linked to the presence of glucoerucin. One of the main products of this glucosinolate is erucin, a biologicallly active isothiocyanate recently recognized as a hydrogen sulfide (H_2_S) donor. In this work, an *Eruca sativa* extract has been obtained from a defatted seed meal (DSM), achieving a powder rich in thiofunctionalized glucosinolates, glucoerucin, and glucoraphanin, accounting for 95% and 5% of the total glucosinolate content (17% on a dry weight basis), associated with 13 identified phenolic acids and flavonoids accounting for 2.5%. In a cell‐free model, *Eruca sativa* DSM extract slowly released H_2_S. Moreover, this extract promoted significant hypotensive effects in hypertensive rats, and evoked dose‐dependent cardioprotection in in vivo model of acute myocardial infarct, obtained through a reversible coronary occlusion. This latter effect was sensitive to blockers of mitochondrial KATP and Kv7.4 potassium channels, suggesting a potential role of these mitochondrial channels in the protective effects of *Eruca sativa* DSM extract. Accordingly, *Eruca sativa* DSM extract reduced calcium uptake and apoptotic cell death in isolated cardiac mitochondria. Taken together, these results demonstrate that *Eruca sativa* DSM extract is endowed with an interesting nutraceutical profile on the cardiovascular system due to, at least in part, its H_2_S releasing properties. These results pave the way for future investigations on active metabolites.

## 1 INTRODUCTION

*Eruca sativa* Mill, a synonym of *E. vesicaria* subsp. *sativa* (Miller) Thell, is the only taxon of *Eruca vesicaria* (L.) Cav. to be cultivated worldwide. It is a diploid annual or biennial herb belonging to *Brassicaceae* family and in Europe, it is known as salad rocket (Great Britain), salatrauke (Germany), oruga or eruca (Spain), roquette (France), rucola (Italy), and while in the USA, it is frequently reported as arugula. *E. sativa* sel. Nemat (Italy) is a selection that produces a bigger seed size than *E. vesicaria*, a difference that permits to classify Nemat as an industrial oleaginous crop ready to be cultivated by agricultural mechanization, so that it can be considered for industrial biorefinery strategies. It spontaneously grows in the Mediterranean basin with different accessions and is cultivated as leaf vegetable or for oilseeds in Northern America, Europe, and Asia (Mirzabe, Hajiahmad, & Asadollahzadeh, 2021).

In Western countries, *E. sativa*'s leaves are used as a salad or garnish and they are sold in both processed and fresh markets, where it has become very popular as “baby leaf” greens over the last two decades (Bell & Wagstaff, 2019; Di Gioia, Avato, Serio, & Argentieri, 2018). In India and Pakistan, special ecotypes of *E. sativa* are widely used as oliseed, forage, and fodder crops (Bhandari & Chandel, 1996).

As regards the beneficial effects in humans, *E. sativa* is known since Roman times for its aphrodisiac properties and other biological activities, including diuretic, stomachic, carminative, and to alleviate abdominal discomfort and improve digestion (Khan & Khan, 2014). Furthermore, this plant possesses anti‐secretory, cytoprotective, and anti‐ulcer activity against experimentally induced gastric lesions (Khan & Khan, 2014). Fuentes and colleagues highlighted anti‐platelet and anti‐thrombotic activity mediated through the inhibition of NF‐kB pathway and then through a reduced release of inflammatory mediators (Fuentes, Alarcón, Fuentes, Carrasco, & Palomo, 2014).

All the reported health benefits provided by *E. sativa* may be ascribed to the presence of different phytochemicals in its tissues. 4‐(methylthio)‐butyl‐GSL, glucoerucin, is the main GSL present in *E. sativa* seeds, together with its oxidized analogue 4‐methylsulfinylbutyl‐GSL, glucoraphanin (Franco et al., 2016). *E. sativa* seeds also contain polyphenols (Sarwar, Kaur, Jabbar, Javed, & Athar, 2007), flavonoids, and carotenoids (Barillari et al., 2005).

Due to the presence of the high amount of phenolic compounds and GSLs, extract from *E. sativa* seeds is considered a rich source of antioxidants and anti‐inflammatory agents (Villatoro‐Pulido et al., 2012).

In particular, *E. sativa* extract inhibits COX2, TLR4, NLRP3 inflammasome pathways, and pro‐inflammatory cytokines and the activation of apoptotic mechanisms in murine motor neurons exposed to the culture medium of lipopolysaccharide‐stimulated murine macrophage cell lines (Gugliandolo et al., 2018).

Recently, *E. sativa* DSM has been demonstrated to be effective in the management of neuropathic pain, in an experimental model of diabetes. Glucoerucin has been identified as hydrogen sulfide (H_2_S) donor moiety and responsible, at least in part, for these beneficial effects (Lucarini et al., 2019).

In humans, H_2_S has been recognized as a gasotransmitter deeply involved in cardiovascular homeostasis and it is implicated also in regulating metabolism, cognitive, respiratory, and reproductive functions (Martelli et al., 2012); whereas in plants, it acts as an antioxidant, improves the performance of the organism under stress conditions and plays significant roles in several physiological processes, including seed germination, morphogenesis, photosynthesis, and flowering (Hancock & Whiteman, 2014). Notable, erucin, likewise glucoerucin, has been demonstrated to promote a slow release of H_2_S in cell‐free and cell‐based models (Citi et al., 2014; Martelli et al., 2020).

Recently, *E. sativa* DSM showed hypoglycaemic and anti‐obesity effects in a rodent model of metabolic disorder (Piragine et al., 2021). Moreover, aerial parts *E. sativa* extract produced endothelium‐mediated vasorelaxation in normotensive conditions, probably due to activation of muscarinic receptors; on the other hand, in hypertensive conditions, an endothelium‐independent vasorelaxation was observed (Salma, Khan, & Shah, 2018).

Anyway, the effects of *Brassicaceae* deriving compounds on the cardiovascular system still need to be fully elucidated; therefore, the main goal of this work was the production and characterization of an *E. sativa* DSM extract, and the evaluation of its implications on the cardiovascular system.

## 2 MATERIALS AND METHODS

### 2.1 Plant material and preparation of Eruca sativa DSM extract

*Eruca sativa* Mill. sel. NEMAT (Lazzeri, Errani, Leoni, & Venturi, 2004) was cultivated during the 2018 season, within a plot with a size of around 1,100 m^2^. The cultivation was carried out at an experimental farm located at Budrio (Bologna) in the Po Valley area (Emilia Romagna region, 44°32′00” N; 11°29′33″ E, altitude 28 m a.s.l.). The field was fertilized by a tertiary fertilizer NPK (11–11‐18) at a dose of 700 kg ha^−1^. *E. sativa* seeds were harvested using a plot combine, cleaned with a fixed Small‐Scale Threshing Equipment (Cicoria Srl, Italy), and finally air‐dried at room temperature. *E. sativa* seeds were defatted by a small continuous seed crusher machine (Bracco Company model Elle.Gi type 0.90, Italy) as previously described (Lucarini et al., 2019) and defatted seed meal (DSM) was characterized for moisture; residual oil, using Soxhlet extraction method with hexane as a solvent; GSL, and phenolic acids and flavonoids according to section 2. The characterized *E. sativa* DSM was ground to 500 μm grain size by the Ultra Centrifugal Mill ZM200 (Retsch GMBH, Germany) and 500 g of homogeneous DSM was extracted in 6 L 70% ethanol for 10 min at 80°C with an Ultra‐Turrax homogenizer (IKA‐Werk, Staufen, Germany). The extract was cooled in the ice bath and then centrifuged at 25,900*g* for 30 min at 10°C. The supernatant was reduced about three‐fold at 40°C, stored at −20°C for 48 hr, centrifuged, and finally lyophilized (DLAB 500, Italian Vacuum Technology, Italy).

### 2.2 HPLC analysis of Desulfo‐Glucosinolates in Eruca sativa DSM extract

For GSL analysis (EN ISO 9167:2019; Franco et al., 2016), 40–50 mg fine‐powdered lyophilized *E. sativa* DSM extract was diluted in 4 ml pure water and 1 ml of the diluted extract and filtered extract (cellulose acetate 0.45 μm syringe filter) was loaded onto a mini‐column filled with DEAE Sephadex A‐25 anion‐exchange resin (GE Healthcare, Germany), conditioned with 25 mM sodium acetate buffer (pH 5.6). 1 ml of the diluted extract was loaded onto a mini‐column filled with DEAE Sephadex A‐25 anion‐exchange resin (GE Healthcare, Germany), conditioned with 25 mM sodium acetate buffer (pH 5.6). After washing with the same buffer (3 ml), sulfatase (200 μL, 0.35 U ml^−1^), purified from *Helix pomatia* (Sigma Aldrich, USA) according to ISO 9167‐1 (EN ISO 9167:2019), was loaded onto the mini‐column, which was then left overnight at 30°C. The desulfated GSLs were finally eluted with four 1 ml portions of ultrapure water and water was allowed to drain after each addition. The desulfated GSLs were detected by HPLC‐UV monitoring their absorbance at 229 nm and identified with respect to their retention times and UV spectra according to our library (Franco et al., 2016). Their amounts were estimated using isolated sinigrin as an internal standard. Sinigrin was isolated as previously reported at HPLC purity >99% and stored at −20°C until required; the response factor for desulfated glucoerucin and glucoraphanin was according to Wathelet et al. (Wathelet et al., 2004). Each dilution of the powdered extract and analysis was performed in triplicate. GSL storage stability of the *E. sativa* DSM extract was conducted for a sample of extract kept at −20°C for about 1 year storage. GSL profile was analyzed at about 1, 2, 3, and 12 months from the date of lyophilisation, as above described.

### 2.3 HPLC analysis of phenolic acids and flavonoids in E. sativa DSM extract

Phenolic acids and flavonoids were analyzed by HPLC (1,290 UHPLC series Rapid Resolution; Agilent Technologies, USA), with a diode array detector. 500 mg of *E. sativa* DSM was extracted twice with 5 ml methanol/ 1 N HCl (85:15) solution by 20 min sonication, with nitrogen gas introduced into the tubes, and centrifugation (4,500 rpm, 10 min, 4°C). Supernatants were recovered and stored at −20°C to precipitate the large molecules. For soluble conjugated phenolic compounds, 5 ml of the methanolic extracts was digested with 6 ml 4 N NaOH in a sonication bath for 10 min. The solutions were then brought to pH 2 with 6 M HCl and extracted twice with 10 ml of diethyl ether/ethyl acetate (1:1 v/v). The extracts were clarified for centrifugation, evaporated, resuspended in 2 ml of mobile phase (phosphoric acid 10^−3^ M / acetonitrile 95:5 v/v), and filtered with a 0.22 μm PTFE membrane before HPLC analysis. The insoluble bound phenolics were obtained from the solid residue of methanolic extracts and were further digested in 12 ml 4 N NaOH in sonication bath for 30 min, vortexing every 5 min. After centrifugation, the supernatant was brought to pH 2 with 6 N HCl and extracted twice with 10 ml of diethyl ether/ethyl ether (1:1 v/v). After separation, the organic phase was clarified for centrifugation, evaporated, resuspended in 2 ml of mobile phase (phosphoric acid 10^−3^ M / acetonitrile 95:5 v/v), and analyzed by HPLC. The final soluble and insoluble phenolic acid extracts were also analyzed for flavonoids. 500 mg of *E. sativa* extract was completely dissolved in methanol/1 N HCl (85:15) solution for soluble phenolic acids and flavonoids extraction. Phenolic acids and flavonoids chromatographic separation was carried out with a 50 mm × 2.1 mm × 1.8 μm column (Eclipse Plus‐C18 RRHD; Agilent). The temperature of the column oven was set at 40°C. The flow rate of the mobile phase was 0.3 ml min^−1^, and the injection volume was 1 μl. The mobile phase was 10^−3^ M phosphoric acid (solution A), acetonitrile (solution B). The gradient, in terms of eluent B was: 0–2 min, 5% B (isocratic); from 2 to‐4.5 min at 10% B; at 5.5 min, 15% B; at 9 min, 35% B; at 11 min, 55% B; from 12 min to 13 min at 70% B (isocratic); after each analysis, the initial mobile phase conditions were set, and the system was allowed to equilibrate for 3 min. Detection wavelengths were 280 nm and 320 nm. For peak quantification, calibration curves of the following standards (Sigma‐Aldrich, Saint Louis, MO, USA) were constructed: gallic acid, protocatechuic acid, *p*‐hydroxybenzoic acid, vanillic acid, syringic acid, and vanillin (between 5 and 40 mg L^−1^); caffeic acid, *p*‐coumaric acid, sinapic acid, *trans*‐cinnamic acid, luteolin, vitexin, apigenin, naringenin, rutin, and quercetin‐3 glucoside (between 2.5 and 20 mg L^−1^). The correlation coefficient for each analyte was in the range (0.97–0.99) (see Figure S1 supplementary materials). Sample compounds were identified based on the retention times and confirmed by their spectra in comparison with standards and were quantified by comparing their peak areas with those of standard curves.

### 2.4 Amperometric approach

The H_2_S‐generating property of *E. sativa* DSM extract has been evaluated by an amperometric approach, through an Apollo‐4,000 Free Radical Analyzer (WPI) detector and H_2_S‐selective mini‐electrodes at room temperature as reported previously (Testai et al., 2016). Briefly, a “PBS buffer 10×” was prepared (NaH_2_PO_4_·H_2_O 1.28 g, Na_2_HPO_4_·12H_2_O 5.97 g, and NaCl 43.88 g in 500 ml of H_2_O) and stocked at 4°C. Immediately, before the experiments, the “PBS buffer 10×” was diluted in distilled water (1:10) to obtain the assay buffer (AB); pH was adjusted to 7.4. The H_2_S‐selective minielectrode was equilibrated in 10 ml of the AB, until the recovery of a stable baseline. Then, 100 μl of a dimethyl sulfoxide (DMSO) solution of *E. sativa* DSM extract was added at the final concentration of 1 mg ml^−1^. The generation of H_2_S was observed for 30 min. In order to explore the contribution of thiol groups, naturally present in the biological environment, in the release of H_2_S, 4 mM L‐cysteine was added, before the extract application.

#### 2.4.1 Data analysis

The correct relationship between the amperometric currents (recorded in pA) and the corresponding concentrations of H_2_S was determined by opportune calibration curves with applying increasing concentrations of NaHS (1, 3, 5, and 10 μM) at pH 4.0. The lowest limit of reliable quantitative determination was 0.3 μM. The vertical bars represent the standard error (n = 9).

### 2.5 In vivo systolic blood pressure values recordings

All the procedures involving animals were carried out following the guidelines of the European Community Council Directive and in accordance with the Code of Ethics of the World Medical Association (Declaration of Helsinki, EU Directive 2010/63/EU for animal experiments). The experiments were authorized by the Ethical Committee of the University of Pisa and by the Italian Ministry of Health (authorization number 751/2018‐PR).

The effects of *E. sativa* DSM extract and its vehicle (DMSO 1 ml kg^−1^) on blood pressure were tested on 12‐week‐old male normotensive, spontaneously hypertensive rats (SHR) and rats in which the hypertension was induced by the administration of a single i.p. injection of phenylephrine (PE) 150 μg kg^−1^ (300–350 g). Briefly, all the rats were anaesthetized with sodium thiopental 60 mg kg^−1^ i.p. and after the administration of the anesthetic drug, they were kept on a heated platform (about 30°C) for 20 min to induce slight vasodilation of the tail artery, in order to allow an easier recording of the basal systolic blood pressure by the “tail‐cuff” method by a BP recorder (BP‐2000 Blood Pressure Analysis System, Series II, Visitech System, Apex NC, USA). The basal level of systolic blood pressure (Psys) was recorded for 20 min, at 5 min intervals. Then, for rats in which hypertension was pharmacologically induced, and after the PE administration, the rise of systolic blood pressure values was monitored for 20 min every 5 min and, at the reaching of stable levels of hypertension, the *E. sativa* DSM extract was administered as following. Briefly, *E. sativa* DSM extracts 10 mg kg^−1^, 100 mg kg^−1^, or the corresponding vehicle, were administered i.p. to different groups (normotensive, SHR, PE‐hypertensive rats). Starting from the administration of the tested compounds, the Psys values were recorded, for 2 hours after 10, 30, 60, 90, and 120 min. Rats were maintained under anesthesia for all experiments, in order to avoid interferences correlated to the stress of experimental procedures.

#### 2.5.1 Data analysis

Basal Psys was expressed as a mean of the four measurements carried out in each rat before the administration of tested compounds. Change in systolic blood pressure, recorded after ES extract or vehicle administration, was expressed as a percentage of the basal Psys and also calculated as the mean value of the five recordings (at 10, 30, 60, 90, and 120 min) carried out after the drug administration. Blood pressure measurements were carried out in 6 animals/group (*n* = 6 for each treatment both in normotensive and SHR groups).

All the experimental data were analyzed by a computer fitting procedure (GraphPad Prism 6.0, La Jolla, CA, USA) and expressed as mean ± SEM of at least six independent experiments. ANOVA and and Bonferroni's post‐test were selected as statistical analyses,and *p* < .05 was considered representative of significant statistical differences.

### 2.6 In vivo acute myocardial infarct

12‐week‐old male Wistar rats (300–320 g) were used accordingly with DL26/2014 (protocol number 909/2016‐PR, 04/10/2016). Two hours before the experimental procedures, animals received an i.p. injection (about 0.35 mL) of *E. sativa* DSM extract (10, 50 or 100 mg kg^−1^) or vehicle (DMSO).

Then, rats were anesthetized with sodium thiopental (70 mg kg^−1^, i.p.) and the experimental protocol for coronary occlusion–reperfusion was performed as previously described (Testai et al., 2016). The acute infarct protocol consisted of 30 min occlusion/120 min reperfusion; successful occlusion was confirmed by observing regional cyanosis downstream of the ligature, and by ST elevation in the ECG recording. In a group of animals, the thread was inserted without ligation (sham). In another experimental set, the potassium channel blockers, 5‐decanoic acid (5‐HD)10 mg kg^−1^ or XE991 2 mg kg^−1^, selective inhibitors of mitoKATP and mitoKv7, respectively, were i.p. administered 20 min before the administration of *E. sativa* DSM extract (100 mg kg^−1^). A group of vehicle‐pretreated animals was submitted to an ischemic preconditioning (IPC) procedure, achieved by two cycles of 5 min occlusion/10 min reperfusion, followed by 30 min coronary occlusion and 120 min reperfusion, and it was used, according to literaute, as cardioprotective protocol.

At the end of reperfusion, rats were euthanized by an overdose of sodium thiopental, then hearts were quickly excised, mounted on a Langendorff apparatus (Radnoti, Monrovia, CA, USA) and perfused for 10 min with Krebs solution at 37°C to wash out the coronary blood vessels. Then, left ventricular tissue was dried, frozen at −20°C, and cut into 4–5 transverse slices from the apex to the base of equal thickness (about 2 mm). The slices were then incubated in a TTC solution in a phosphate buffer (pH 7.4) at 37°C for 20 min. TTC reacts with NADH in the presence of dehydrogenase enzymes, to form a formazan derivative, which stain the viable cells with intense red color. Then, the slices were fixed overnight in 10% formaldehyde and finally photographed. In the viable area, red‐stained viable tissue was easily distinguished from the white‐unstained necrotic tissue.

#### 2.6.1 Data analysis

The ischemic area (Ai) was planimetrically evaluated using an image analyzer program (The GIMP 2). The infarct size was calculated as a percentage of the whole left ventricle area (A_LV_).

All the values were expressed as a mean ± SEM for six different experiments. Data were statistically analyzed by ANOVA and Bonferroni's post‐test. P‐values lower than 0.05 were considered as indicative of significant differences.

### 2.7 Calcium‐uptake evaluation on isolated cardiac mitochondria

#### 2.7.1 Isolation procedure

Rat cardiac mitochondria were isolated by differential centrifugation in accordance with the authorization registered to the Italian Minister of Healthy (protocol number 45,972, September 21, 2016) and as previously described (Testai et al., 2016), with minor modifications. Briefly, 12 week‐old male Wistar rats (300–320 g) were sacrificed by sodium thiopental overdose, hearts were immediately removed and placed in an ice‐cold isolation buffer (IB1, composition mM: sucrose 250, Tris 5, EGTA 1, pH 7.4 adjusted with HCl). The atria were removed, and the ventricular tissue was finely minced with surgical scissors (about 2 mm^3^ pieces) and homogenized by using an Ultra‐Turrax homogenizer (20 mL of isolation buffer per heart, IKA1‐Werke GmbH & Co., Staufen, Germany).

Three homogenization cycles (each of 20 s) were performed on ice, and then the suspension was centrifuged at 1075 g for 3 min at 4°C (EuroClone, Speed Master 14 R centrifuge, Milano, Italy). The resulting supernatant was centrifuged at 11,950 g for 10 min at 4°C. The pellet containing the mitochondrial fraction was further re‐suspended in the isolation buffer (without EGTA, IB2) and centrifuged at 11,950 g for 10 min at 4°C; this step was repeated once more. The final mitochondrial pellet was re‐suspended in a minimal volume of 400 ml of the IB2 and stored on ice throughout the experiments, which were performed within 2 h. Mitochondrial protein concentrations were determined using the usual Bradford reaction.

#### 2.7.2 Calcium‐uptake measurement

Mitochondrial calcium‐uptake was measured by potentiometric technique, as previously described (Testai et al., 2016). In particular, the changes of the calcium concentration in the medium (i.e., extra‐mitochondrial calcium) were continuously measured with a calcium‐selective mini‐electrode, coupled with a reference electrode (WPI, FL, USA), using a data acquisition software (Biopac Systems Inc., Goleta, CA, USA). In order to correlate the potentiometric measurements (in mV) with the corresponding concentrations of calcium ions in the solution, calibration curves were generated before each experiment, by using known concentrations of CaCl_2_. Mitochondria (1 mg protein ml^−1^) were added, under gentle stirring, to the IM plus CaCl_2_ 100 μM in the presence of vehicle (DMSO 1%) or *E. sativa* DSM extract (10, 30 and 100 μmol L^−1^ of total GSLs). After the addition of mitochondria, the maximal decrease of the calcium concentration in the medium, related to its accumulation in the mitochondrial matrix, was measured.

#### 2.7.3 Data analysis

Mitochondrial calcium uptake was evaluated by measuring the reduction of the extra‐mitochondrial CaCl_2_ concentration, following the addiction of mitochondria into a calcium‐rich buffer. All data are expressed as a mean ± SEM. Data were statistically analyzed by ANOVA and Bonferroni's post‐test. *p*‐values lower than .05 were considered as indicative of significant differences. Each result was obtained with mitochondria isolated from the hearts of six different animals.

## 3 RESULTS

### 3.1 Characterization of the E.sativa DSM extract

**FIGURE 1:**
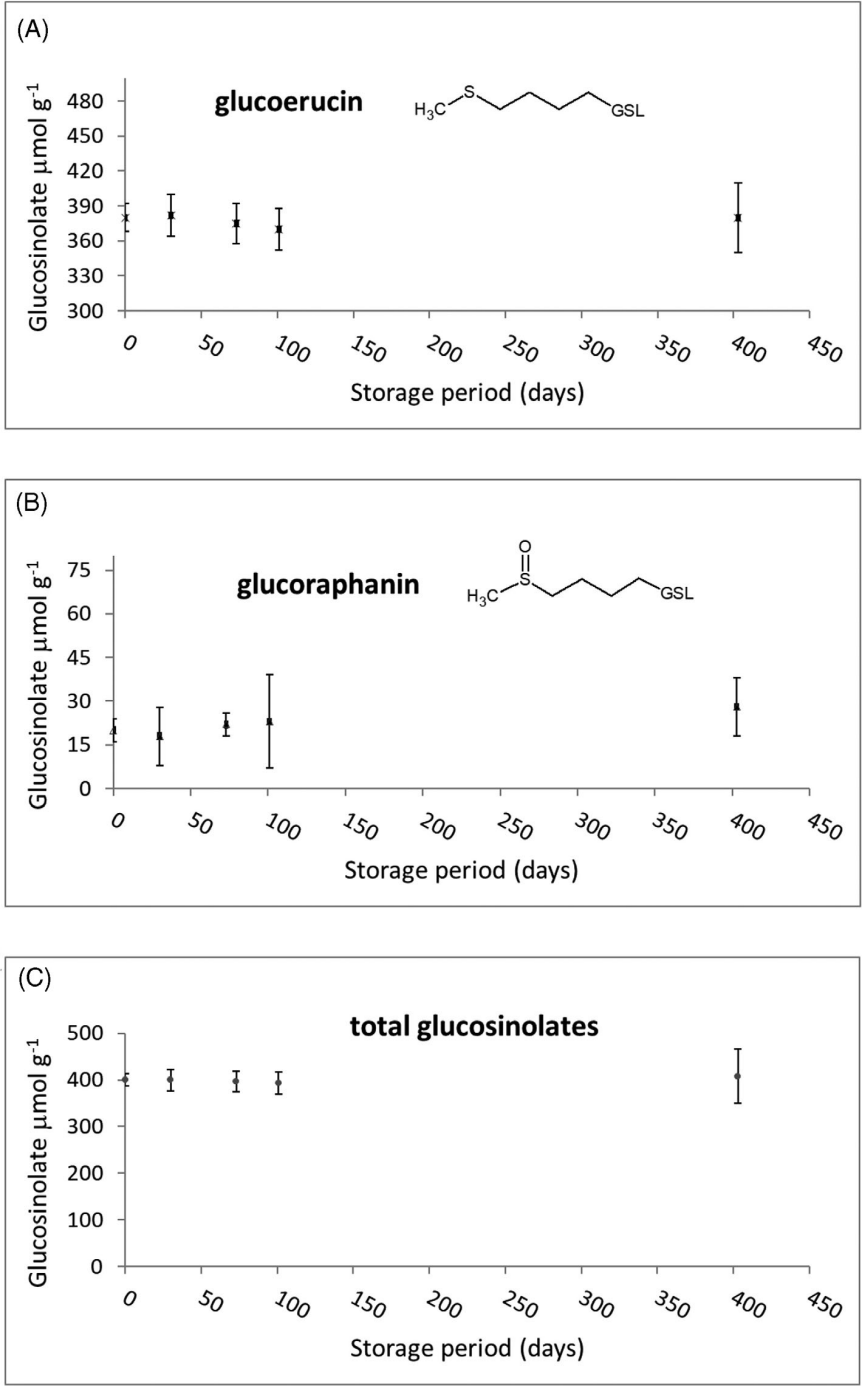
Glucoerucin (a), glucoraphanin (b) and total glucosinolate (c)(GSL), glucoraphanin (GRA) and glucoerucin (GER) content in E. sativa defatted seed meal (DSM) lyophilized extract for a period of storage of about 12 months at −20°C and means ± standard deviation (SD) (*n* = 3) were presented

Starting from an *E. sativa* DSM characterized by 8.6% relative moisture, 16.9 ± 0.8% residual oil, and 125 ± 3 μmol g^−1^ total GSLs, with 92.8% glucoerucin and the remaining glucoraphanin, 95 g of brown powder containing 400 ± 15 μmol g^−1^ total GSLs were obtained. Total GSLs in *E. sativa* lyophylized extract corresponded to 20 ± 1 μmol g^−1^ glucoraphanin and 380 ± 12 μmol g^−1^ glucoerucin, accounting for a 0.9% and a 16% on a weight basis, respectively. The final recovery in GSLs, referring to initial GSL content in *E. sativa* DSM, after a single ethanol extraction attested to 60%. For about 12 months (403 days) under chilled storage conditions (−20 ± 2°C), the *E. sativa* DSM lyophilized extract seemed homogeneous without sign of color changes and exhibited only minor changes in GSL content (Figure 1).

The polyphenol complete characterization was reported in Table 1 and Table 2, and the total soluble polyphenol fraction identified accounted for 2.5% on a weight basis in lyophilized *E. sativa* DSM extract. Flavonoids (57.6%) were the most representative of the soluble polyphenols fraction in *E. sativa* DSM, followed by phenyl carboxylic (24.5%) and phenyl cinnamic (17.9%) acids; the insoluble fraction was almost totally represented by the phenyl cinnamic acid and aldehyde group (93.7%). In the *E. sativa* DSM extract, the group of phenyl carboxylic acids was the most representative (58.9%) of the soluble polyphenols fraction, followed by phenyl cinnamic acids (28.9%) and flavonoids (12.2%). Gallic acid is the main component of soluble phenyl carboxylic acids and aldehydes both in *E. sativa* DSM and in *E. sativa* DSM extract, 84% and 57% respectively, while sinapic acid was the main component within the soluble and insoluble fractions of phenyl cinnamic acids, in *E. sativa* DSM and in the soluble fraction of phenyl cinnamic acids in *E. sativa* DSM extract. Regards to flavonoids group, apigenin and naringenin resulted as the most abundant in soluble and insoluble fraction of DSM, respectively, while luteolin was the most abundant component in *E. sativa* lyophilized extract.

**TABLE 1:** Soluble conjugated and insoluble bound phenolic acids and aldehyde (μg g^−1^) in *E. sativa* defatted seed meal (DSM) and in *E. sativa* defatted seed meal lyophilized extract (ES). Average values ± standard deviation (*n* = 3) are shown

	Gallic acid	Protocatechuic acid	*p*‐hydroxybenzoic acid	Vanillic acid	Syringic acid	Vanillin	Caffeic acid	*p*‐coumaric acid	Sinapic acid	*Trans*‐cinnamic acid
Soluble conjugated phenolic acids and aldehyde										
DSM	1,454 ± 27	29.1 ± 0.2	104 ± 18	10.0 ± 0.9	28 ± 3	114 ± 6	22.0 ± 0.2	130 ± 9	965 ± 59	150 ± 9
ES	8,309 ± 426	69 ± 2.4	189 ± 24	1,102 ± 52	4.3 ± 0.6	4,822 ± 122	422 ± 12	232 ± 12	6,436 ± 286	n.d.
Insoluble conjugated phenolic acids and aldehyde										
DSM	30 ± 24	n.d.	n.d.	n.d.	3 ± 2	2.8 ± 0.2	11 ± 5	66 ± 14	1,215 ± 85	49 ± 12

Abbreviation: n.d. not detected.

**TABLE 2:** Soluble and insoluble flavonoids (μg g^−1^) in *Eruca sativa* defatted seed meal (DSM) and in *E. sativa* defatted seed meal lyophilized extract (ES). Average values ± standard deviations (*n* = 3) are shown

	Luteolin	Vitexin	Apigenin	Naringenin	Rutin
Soluble conjugated flavonoids					
DSM	1,135 ± 81	833 ± 24	1838 ± 188	161 ± 4	105 ± 12
ES	1,614 ± 91	503 ± 18	n.d.	452 ± 30	426 ± 18
Insoluble conjugated flavonoids					
DSM	n.d.	1.4 ± 0.3	17 ± 6	26 ± 3	11 ± 5

### 3.2 E.sativa DSM extract releases H_2_S

**FIGURE 2:**
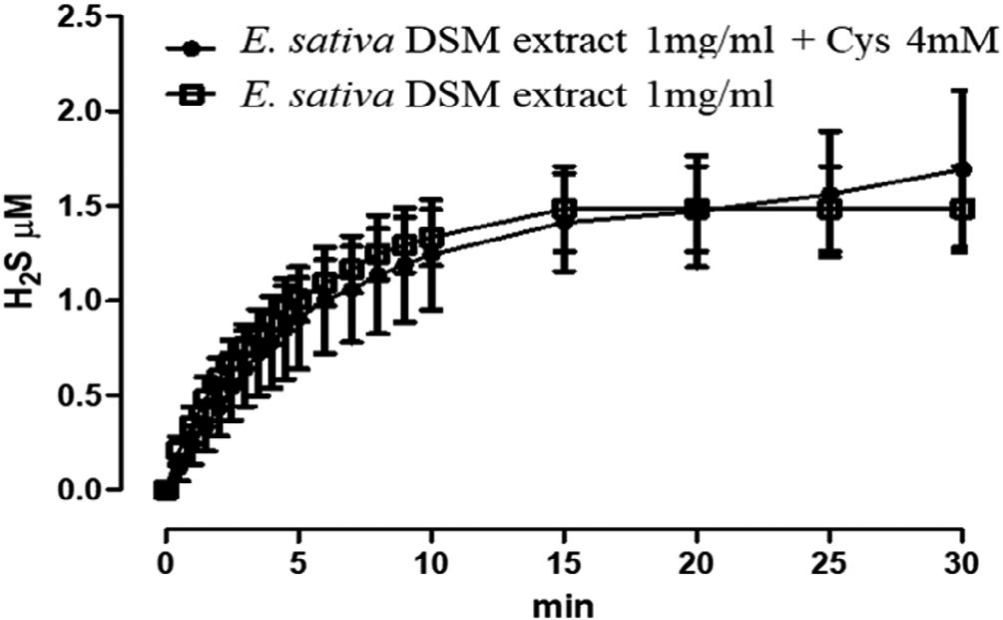
Amperometric recording of the time‐course of H_2_S‐release from *E. sativa* defatted seed meal (DSM) extract in the presence and in the absence of L‐cysteine (Cys 4 mM). The vertical bars indicate SEM

*E. sativa* DSM extract incubated at the concentration of 1 mg ml^−1^, corresponding to 0.4 mM total GSLs, released H_2_S with a slow kinetic both in the presence and in the absence of L‐cysteine (L‐Cys, 4 mM), reaching a stable steady state of about 1,5 μM (Figure 2).

### 3.3 E.sativa DSM extract shows in vivo anti‐hypertensive effects

**FIGURE 3:**
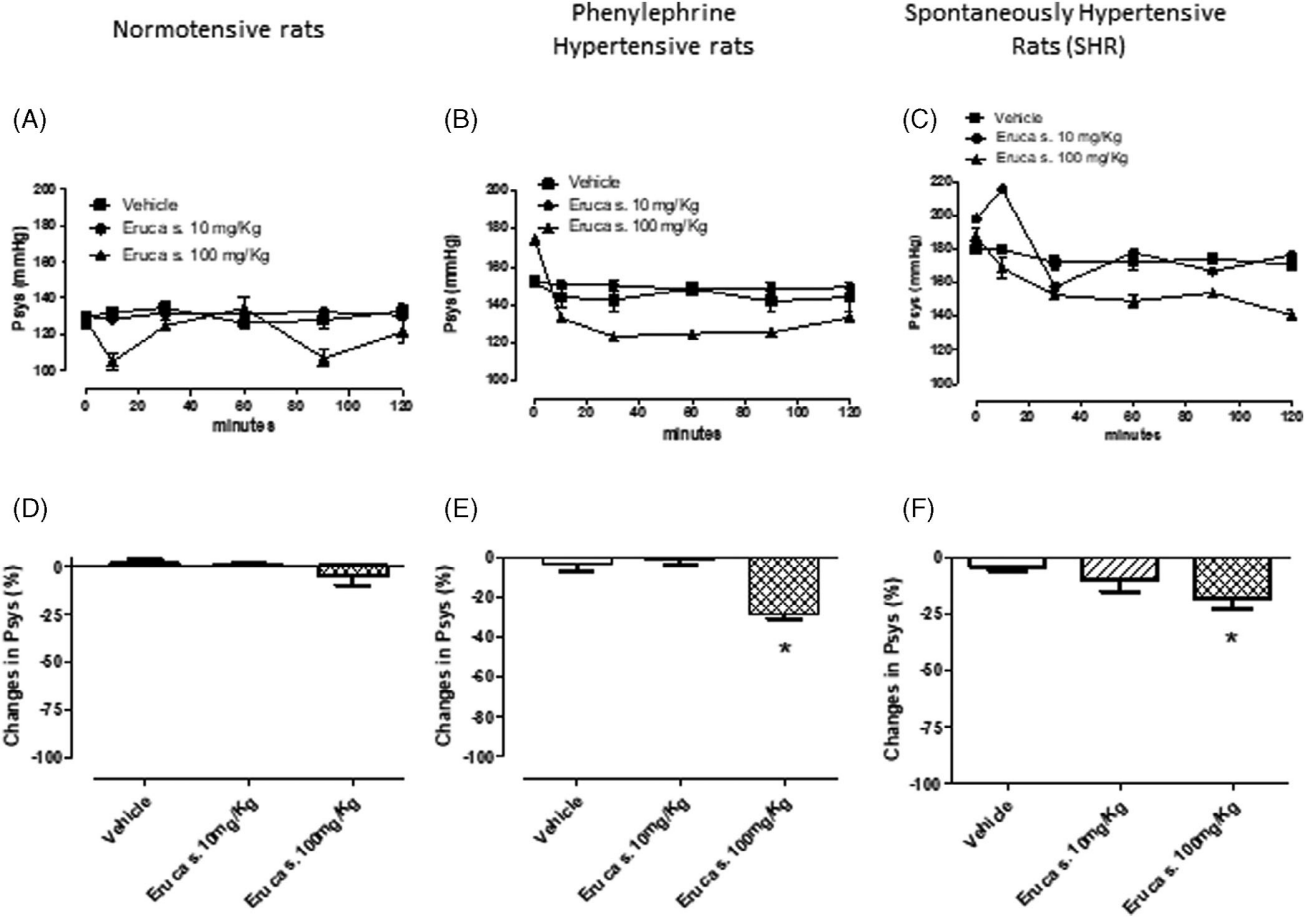
Time‐course of changes in systolic blood pressure values (expressed in mmHg, in the left part of the figure) and mean change in Psys (expressed as a % of the basal Psys values, in the right part of the figure). (a) normotensive rats, (b) PE‐induced hypertensive rats and (c) spontaneously hypertensive rats after i.p. administration of vehicle or *E. sativa* defatted seed meal (DSM) extract at 10 or 100 mg kg^−1^. The symbol * indicates significant differences vs vehicle

*E. sativa* DSM extract administrated in vivo to normotensive rats did not modify the levels of systolic blood pressure; indeed, only a minimal and not significant reduction was observed at the highest dose (100 mg kg^−1^, Figure 3a). In agreement with literature (Martelli, Piragine, et al., 2020; Talmor et al., 1998), both SHR and PE‐treated rats showed Psys values markedly higher if compared with normotensive ones (174 ± 5 mm Hg and 152 ± 2 mm Hg, respectively). Interestingly, in such hypertensive conditions, *E. sativa* DSM extract was able to significantly reduce Psys values; indeed, at the end of recording, a clear decrease of Psys was observed in both types of hypertension (−28 ± 3% in PE‐treated rats and − 18 ± 4 in SHR; Figure 3b, c). The hypotensive efficacy of *E.sativa* DSM extract was found to be comparable to the isothocyanate erucin, used as a positive control (Figure S2 in supplementary materials).

### 3.4 E.sativa DSM extract shows in vivo anti‐ischemic cardioprotection

**FIGURE 4:**
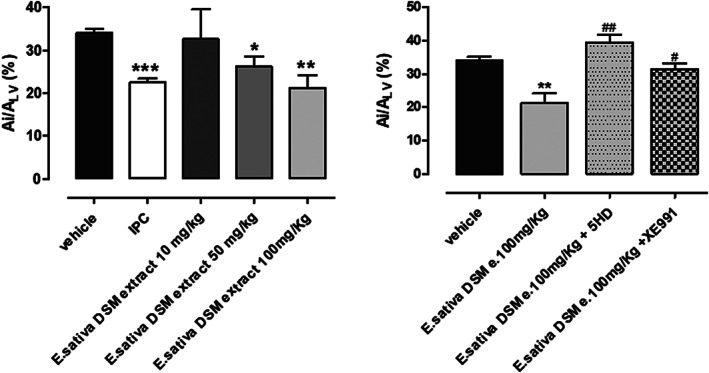
Morphometric quantification of Ai/A_LV_% observed in ventricular slices of rat hearts, after acute myocardial infarction in vivo. (a) After treatment with vehicle or *E. sativa* defatted seed meal (DSM) extract at different doses (10, 50 and 100 mg kg^−1^). (b) After the treatment with *E. sativa* DSM extract 100 mg kg^−1^ in the presence or in the absence of the selective mitoK blockers, mitoKATP and mitoKv7.4, 5HD (10 mg kg^−1^) and XE991 (2 mg kg^−1^), respectively. The symbol * indicates significant differences vs vehicle and the symbol # indicates significant differences vs *E. sativa* DSM extract (100 mg kg^−1^)‐treatment

According to literature, 30 min of coronary occlusion followed to 120 minutes of reperfusion produced a significant injury (Testai et al., 2016), indeed ischemic size was 34 ± 1%; conversely, in the sham group, the amount of injured area was very low (Ai/A_LV_ = 11 ± 4%). An i.p. injection of *E. sativa* DSM extract (10 mg kg^−1^) 2 hours before the experimental procedure did not reduce ischemic area (Ai/A_LV_ = 33 ± 7%. %), while *E. sativa* DSM extract at the doses 50 and 100 mg kg^−1^ significantly contained the ischemic damage (Ai/A_LV_ = 26 ± 2% and 21 ± 3%. %, respectively), similarly to that was observed with IPC procedure (Figure 4a).

The protective effects observed at the highest dose of the extract were almost completely abolished by the pre‐treatment with selective mitoKATP and Kv7 channel blockers, 5HD (10 mg kg^−1^, Ai/A_LV_ = 39 ± 2%) and XE991 (2 mg kg^−1^, Ai/A_LV_ = 31 ± 2%), respectively (Figure 4b).

### 3.5 Effects of E.sativa DSM on mitochondrial calcium movements

**FIGURE 5:**
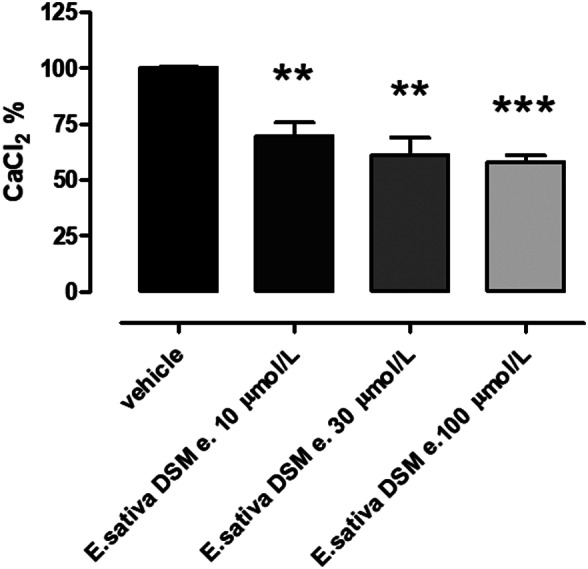
Intra‐mitochondrial uptake of calcium ions (in % vs. 100 μM), following the administration of isolated cardiac mitochondria in the presence of vehicle or *E.sativa* defatted seed meal (DSM) extract at different concentrations. The concentration of the extract is related to glucosinolate title. The symbol * indicates a statistically significant difference vs vehicle

It is well‐known that mitochondria greedily uptake calcium ions. The addition of *E. sativa* DSM extract to cardiac mitochondria produced a concentration‐dependent reduction in the accumulation of calcium (Figure 5).

## 4 DISCUSSION

Vegetables belonging to *Brassicaceae* family are largely consumed even for possible health benefits. They contain high levels of GSLs, which are sulfur‐derivatives able to produce active metabolites, named isothiocyanates, after the hydrolysis mediated by myrosinase enzyme (Martelli, Piragine, et al., 2020). Noteworthy, even if cooking and pH at gastric level can cause denaturation of myrosinase, thioglucosidase activity of gut bacteria can hydrolyze GSLs and ensure the formation of isothiocyanates (Martelli et al., 2014).

To our knowledge, the *E. sativa* seed extract obtained and characterized in this paper is endowed with the highest content of identified molecules and the highest concentration in GSL.

Indeed, other authors reported that *E. sativa* seeds Soxhlet extracted with 95% ethanol contained up to 0.45% glucoerucin and 0.65% total flavonoids (Sarwar et al., 2007). Gugliandolo et al. obtained *E. sativa* seed extract enriched in glucoerucin up to about 4.6% on weight basis, with additional 0.2% (w/w) total flavonoids and 0.016% (w/w) ascorbic acid (Gugliandolo et al., 2018). Recently, an *E. sativa* seed extract obtained in 95% ethanol in an incubator shaker at 30°C for 2 days was reported. GSLs were not analyzed, and probably the extract contained rather high amounts of GSL hydrolysis products, but HPLC analysis of phenolic compounds revealed the presence of 10 phenolic compounds, accounting about 7% on a weight basis, of which the main ones were catechin, caffeic acid, and 3,5‐dicaffeoyl quinic acid (Abdel‐Rahman et al., 2015). Finally, the LC/MS analysis of an ethanol extract of *E. sativa* whole seeds revealed the tentative identification of 39 compounds belonging to sulphur‐containing compounds, flavonoids, phenolic acids and fatty acids, with no further information on quantitative composition (Abd‐Elsalam et al., 2021).

Considering that the isothiocyanate moiety has been recognized as a smart H_2_S‐donor chemical group (Citi et al., 2014; Lucarini et al., 2018; Martelli, Piragine, et al., 2020; Pagliaro, Santolamazza, Simonelli, & Rubattu, 2015; Ritz, Wan, & Diaz‐Sanchez, 2007), we hypothesized that, at least in part, the numerous beneficial effects observed with a *Brassicaceae* enriched diet may be due to the release of this gasotransmitter. In this regards, natural isothiocyanates, that is, erucin and sulforaphane (Citi et al., 2014), can release H_2_S only in the presence of endogenous thiols. Surprisingly, *E. sativa* DSM extract shows an L‐cysteine‐independent H_2_S donation. We hypothesize that probably this behavior can be due to the presence of some substances which may favor the H_2_S release. Among flavonoids founded in *E. sativa* extract, rutin seemed an attractive candidate for an L‐cysteine‐independent release of H_2_S; it was in fact associated to an increase of H_2_S level, with subsequent up‐regulation of the expression of nuclear factor‐E2‐related factor‐2 (Nrf2) and heme oxygenase‐1 (HO‐1) in dorsal root ganglions of diabetic rats (Tian et al., 2015).

Besides the well‐known cancer‐preventive properties, vegetables from *Brassicaceae* family show notable beneficial effects on cardiovascular system. Several clinical and pre‐clinical evidence point out protection induced by some *Brassica* spp; in particular, sulforaphane (isothiocyanate deriving from the GSL glucoraphanin)‐induced expression of detoxification enzymes and several nuclear factors, such as Nrf2, involved in reactive oxygen species elimination and xenobiotic excretion in in vitro models (Ritz et al., 2007). Moreover, rats receiving a supplementation for 14 weeks of 200 mg day^−1^ of dried broccoli sprouts, showed an improvement of antioxidant reserves and lower blood pressure value in SHR (Wu et al., 2004). Likely, we observed that, in hypertensive conditions, *E. sativa* DSM extract has been able to significantly reduce Psys values; such an effect could be due to the main isothiocyanate erucin, hydrolyzed by glucoerucin. Indeed, Martelli and colleagues recently demonstrated that erucin possesses a H_2_S‐donor profile in cell‐free and in cell‐based models and induced clear vasodilating and anti‐hypertensive effects (Martelli et al., 2021; Martelli, Piragine, et al., 2020).

In addition, our results demonstrate that *E. sativa* DSM extract possesses anti‐ischemic effects, accordingly to other *Brassica* extracts, that have recognized protective effects against oxidative stress on cardiomyocytes (Yang, 2018) and in in vivo models of myocardial infarct (Jana et al., 2017).

Moreover, the involvement of mitochondrial potassium (mitoK) channels has been hypothesized, since that specific blockers of mitoKATP and mitoKv7 channels almost completely abolished the cardioprotection. Such a profile further confirms the critical role of mitoK channels and strengthens the hypothesis of the presence of an active compound able to stimulate these ion channels; indeed, a mitoK opener is able to induce a mild depolarization of the cardiac mitochondrial membrane, responsible for a lower driving force for accumulating of calcium ions and then reduces the probability to assemblage and opening of the mitochondrial permeability transition pore (MPTP), preventing ultimately the organelle swelling and cell apoptotic death (Testai, Rapposelli, Martelli, Breschi, & Calderone, 2015).

Although the putative mechanisms of action through which H_2_S may play cardioprotective effects are numerous and include the engagement of antioxidant transcription factors (such as Nrf2), anti‐inflammatory cytokines and other agents, such as nitric oxide, mitoK channels are recognized as an interesting target. Of note, the cardioprotection promoted by NaHS in a model of myocardial I/R injury was significantly reversed by pharmacological blockage of mitoKATP channels (Citi et al., 2018; Sivarajah et al., 2009). The involvement of mitoKATP channels in the cardioprotective effects of H_2_S was also ex vivo and in vivo demonstrated, by using a synthetic H_2_S‐donor isothiocyanate (Testai et al., 2016). Noteworthy, sulforaphane at micromolar concentrations protected both cultured cardiomyoblasts and adult cardiomyocytes from H_2_O_2_‐induced oxidative stress through the modulation of Nrf2 signaling pathway (Corssac et al., 2018).

## 5 CONCLUSIONS

On the basis of the phytochemical characterization of this extract, it is possible to speculate that cardiovascular benefits observed, other than GSL‐derived isothiocyanates, may be due to the contribution of flavonoids and phenolics acids found in the *E. sativa* DSM extract; among these luteolin and sinapic acid are particularly relevant. Very recently, it has been demonstrated that luteolin is able to induce cardioprotective effects in vitro and in vivo through mechanisms involving the activation of Nrf2 and the inhibition of the NF‐kβ pathways (Vashi & Patel, 2021). Furthermore, luteolin has also been reported to be cardioprotective via the phosphatidylinositol‐4,5‐bisphosphate 3‐kinase (PI3K)/Akt pathway and able to increase the phosphorylation of phospholamban (PLB) and sarco/endoplasmic reticulum Ca^2+^‐ATPase (SERCA2a) to protect the heart against acute myocardial ischemia–reperfusion injury (Chin, Qin, May, Ritchie, & Woodman, 2017). Moreover, sinapic acid has been recognized in vitro and in vivo as an antioxidant and cardioprotective agent against ischemia–reperfusion injury (Silambarasan et al., 2015).

Furthermore, Olson et al. highlighted that tea polyphenols may autoxidize, thus becoming pro‐oxidants and favouring the oxidation of H_2_S to polysulfides (H_2_S_n_; where *n* = 2–5) and other sulfoxides (Olson, Gao, & Straub, 2021).

Taken together with our results, this evidence suggests the possibility of synergistic mechanisms between H_2_S released by GSLs and phenolic acids in *E. sativa* extracts, that may contribute to the evidenced benefits on cardiovascular system.

Although an accurate pharmacokinetic analisys will have to be executed, this work demonstrates the benefits of this *E. sativa* DSM extract on the cardiovascular system, suggesting that the daily consumption of this vegetable may be associated to beneficial effects on cardiovascular system and that H_2_S may be an end‐effector of such a nutraceutical profile. Future studies will be focused on the characterization of the pharmacological profile of main constituents, specially glucoerucin and its derivative erucin.

## CONFLICTS OF INTEREST

The authors declare no competing financial interest.

## AUTHOR CONTRIBUTIONS

Conceptualization, V.Ca., L.T. and E.Pa.; Methodology, E.Pa., E.Pi., L.F., R.M., S.S. A.M.; Data Curation, E.Pi., L.F., A.T., A.M. and V.Ci. Writing – Original Draft Preparation, L.T., A.M., V.Ci., and E.Pa.; Writing – Review & Editing, L.D.M., N.P., C.G., V.Ca., A.T.; Supervision, V.Ca., N.P. Project Administration, V.Ca.; Funding Acquisition, L.T., V.Ca., A.M., N.P.

## Supporting information


Figure S1

Figure S2

## Data Availability

Data openly available in a public repository that issues datasets with DOIs
