# Supplementary material for: Cardiovascular benefits of Eruca sativa mill. Defatted seed meal extract: Potential role of hydrogen sulfide
Source: Phytother Res. 2022 Apr 27;36(6):2616–27. doi: 10.1002/ptr.7479 (PMC9320972; doi:10.1002/ptr.7479)

Calibration curves of phenolic acids and flavonoids analysed by HPLC. Data represent arithmetical averages of duplicates measured at injection volumes 1.0 µl. Together, they were fitted by linear regression.

Gallic acid

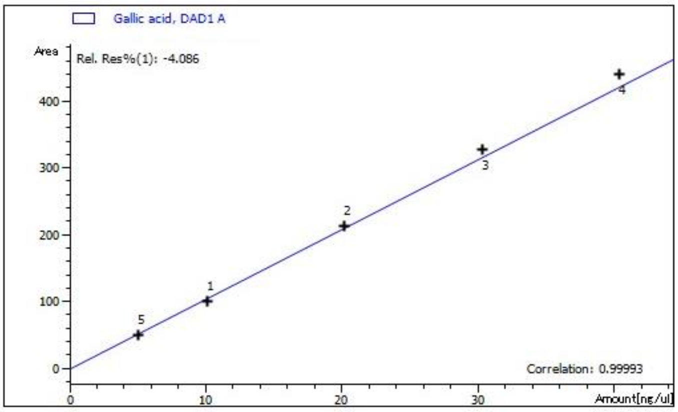

Protocatechuic acid

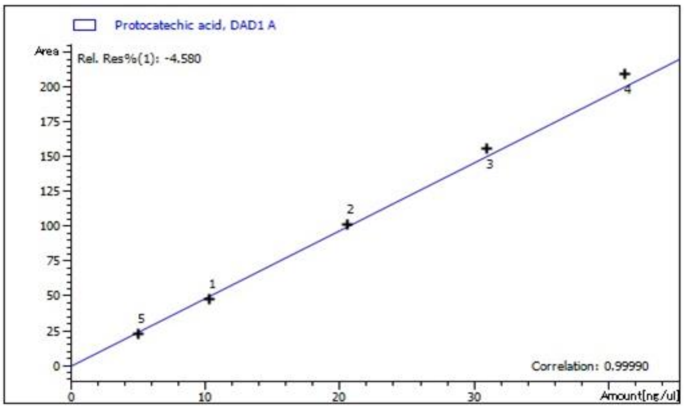

p-hydroxybenzoic acid

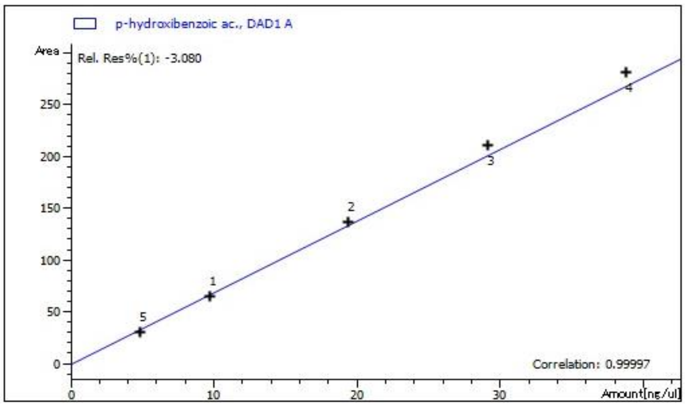

Vanillic acid

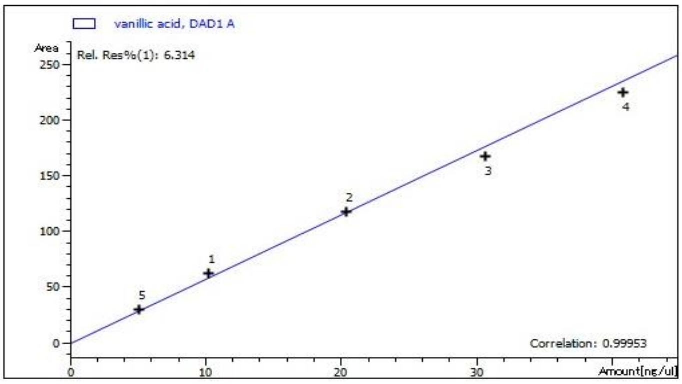

Syringic acid

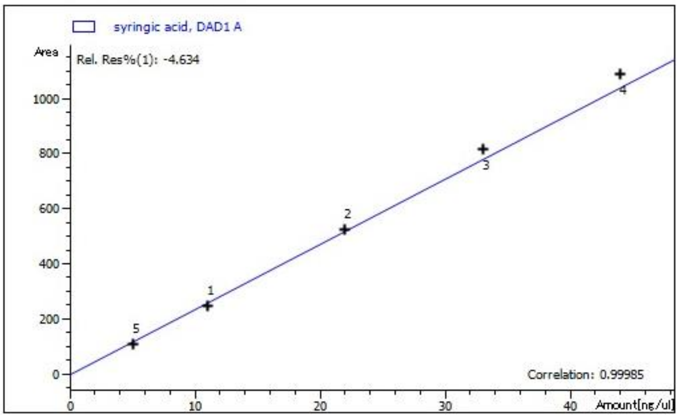

Vanillin

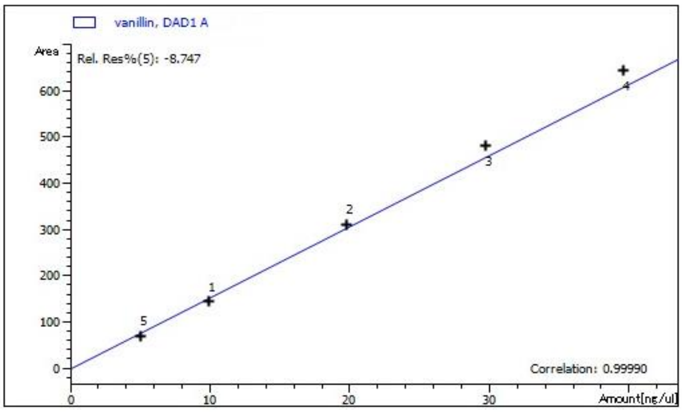

Caffeic acid

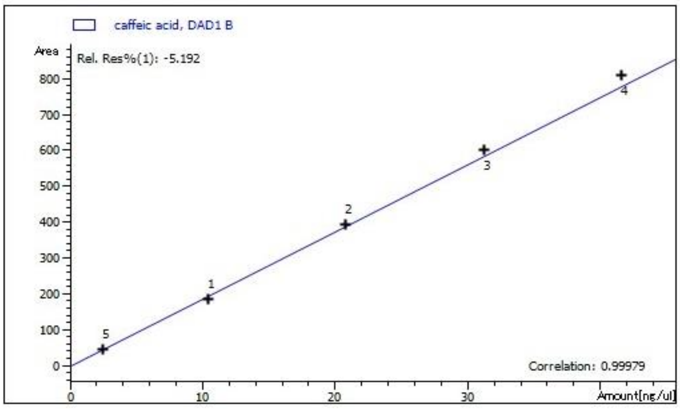

p-coumaric acid

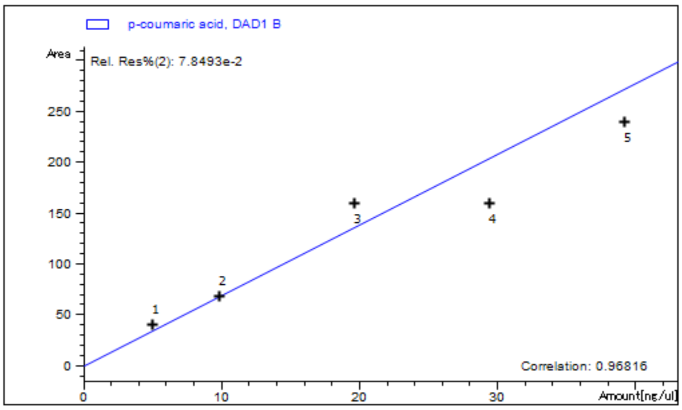

Sinapic acid

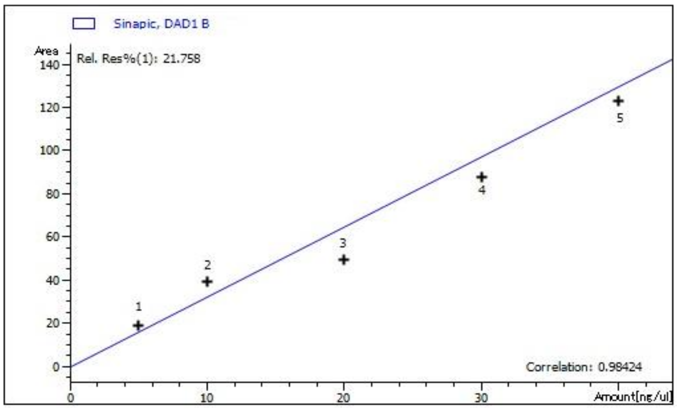

Trans-cinnamic acid

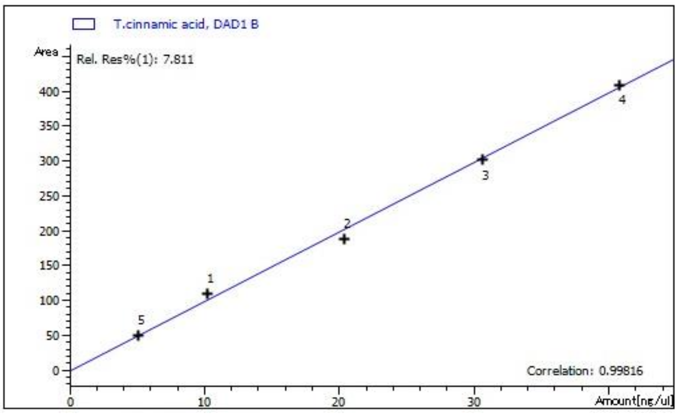

Luteolin

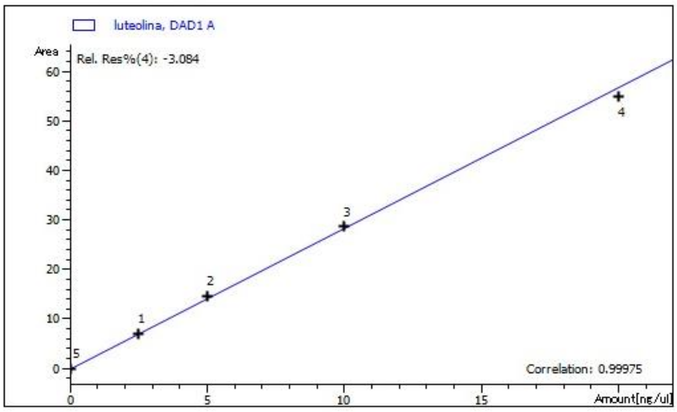

Vitexin

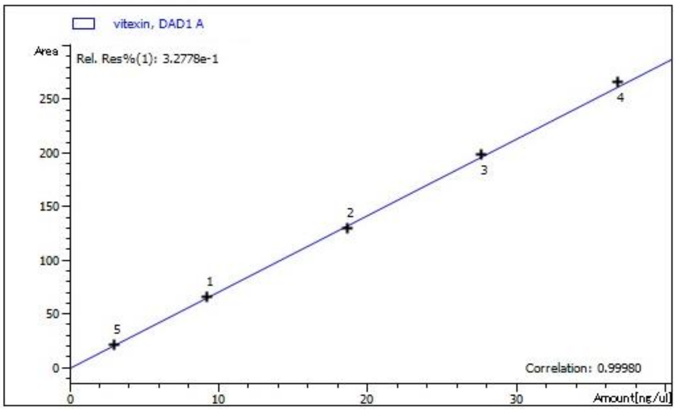

Apigenin

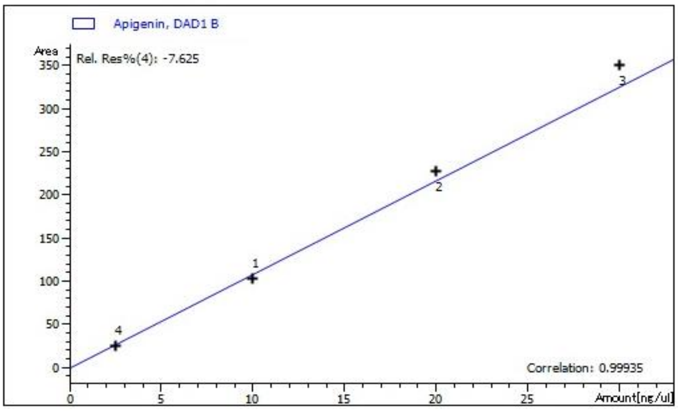

Naringenin

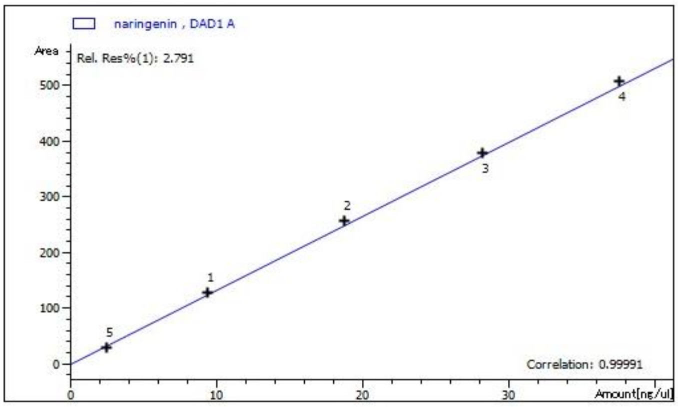

Rutin

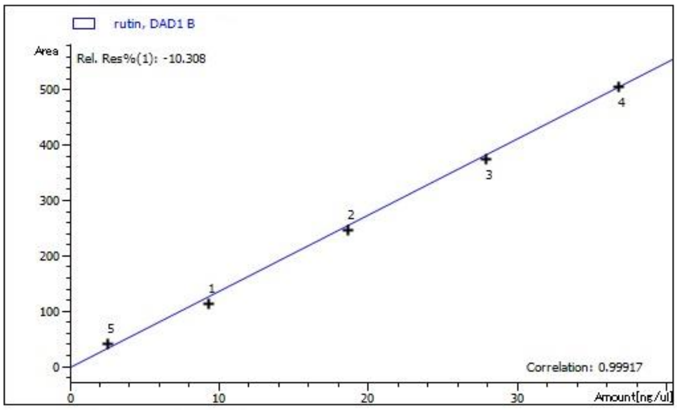

Supplement: Supplementary file 1 — Figure S1 [file PTR-36-2616-s001.pdf]
