# Supplementary figures and images for: Cardiovascular benefits of Eruca sativa mill. Defatted seed meal extract: Potential role of hydrogen sulfide
Source: Phytother Res. 2022 Apr 27;36(6):2616–27. doi: 10.1002/ptr.7479 (PMC9320972; doi:10.1002/ptr.7479)

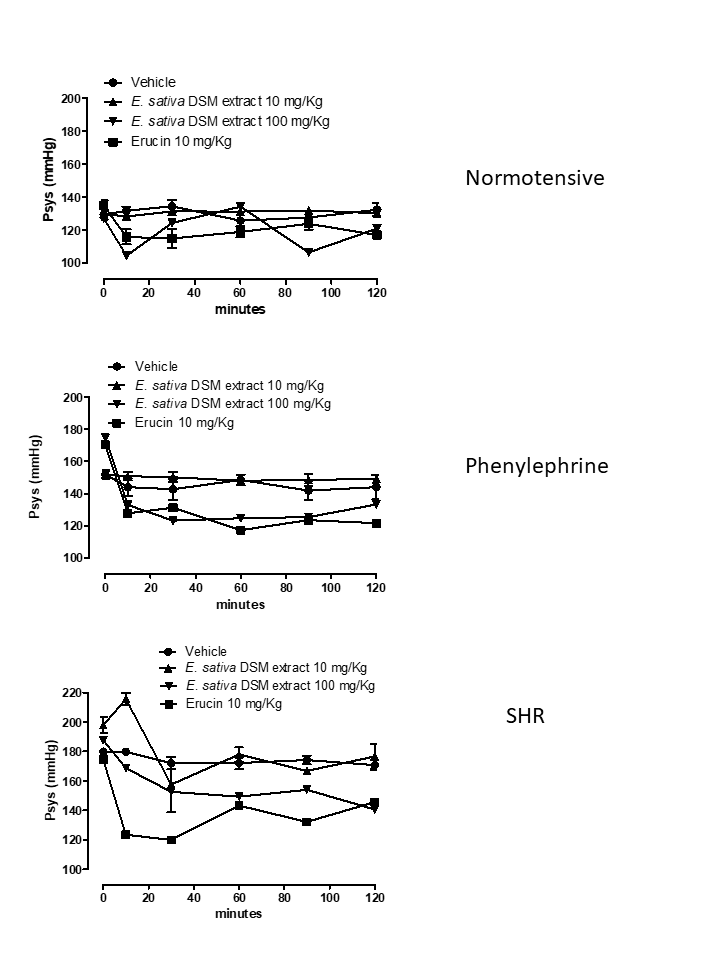

Supplement: Supplementary file 2 — Figure S2 [file PTR-36-2616-s002.tif]
